# Health outcomes, education, healthcare delivery and quality – 3053. Is it necessary to re-evaluate the airway hyperresponsiveness while in the treatment of mild asthma?

**DOI:** 10.1186/1939-4551-6-S1-P222

**Published:** 2013-04-23

**Authors:** Jaechun Lee, Jong Hoo Lee, Miok Kim

**Affiliations:** 1 Pulmonary Medicine and Allergy, Jeju National University, Jeju, South Korea

## Background

Airway hyperresponsiveness (AHR) is one of the typical characteristics of asthma, but its natural course is not known. While asthmatics are under medical treatment, whether their AHRs are present or not is hardly assessed. In mild asthmatics, we investigate the change of AHR in comparison to clinical parameters.

## Methods

Patients diagnosed with asthma, but asymptomatic for more than 3 months while undergoing medical treatment were enrolled. AHR was measured through methacholine bronchial provocation test after a 2-week wash-out period. AHR-negative was defined as PC20 is greater than 25mg/mL. Clinical parameters were compared retrospectively between the AHR-negative and the AHR-positive patients.

## Results

Among 54 patients, 22 (40.7%) were AHR-negative. The considerable factors for the maintenance of AHR is male sex, presence of dyspnea at initial presentation and high dose inhaled corticosteroid plus long acting beta agonist at initial presentation (respectively, *p* < 0.05). Age, symptoms other than dyspnea, blood tests, results of the spirometry, diagnostic methods at presentation and time intervals from diagnosis to follow-up tests showed no difference between AHR-negativity and AHR-positivity. Multivariable analyses failed to show a statistical significance in the two groups.

## Conclusions

In mild asthmatics, about 40% might show no AHR, the clinical remission of the disease. Changing AHR status in mild asthma while undergoing medical treatment is not predictable, so that cessation of regular controller medication might be advocated, and then a reassessment of AHR should be mandatory.

